# Chronic Stress Related to Cancer Incidence, including the Role of Metabolic Syndrome Components

**DOI:** 10.3390/cancers16112044

**Published:** 2024-05-28

**Authors:** An Thanh Pham, Boukje A. C. van Dijk, Eline S. van der Valk, Bert van der Vegt, Elisabeth F. C. van Rossum, Geertruida H. de Bock

**Affiliations:** 1 Department of Epidemiology, University Medical Center Groningen, University of Groningen, 9700 RB Groningen, The Netherlands; b.a.c.van.dijk@umcg.nl (B.A.C.v.D.); g.h.de.bock@umcg.nl (G.H.d.B.); 2 Faculty of Pharmacy, Ton Duc Thang University, Ho Chi Minh City 700000, Vietnam; 3 Department of Research and Development, Netherlands Comprehensive Cancer Organisation (IKNL), 3511 CV Utrecht, The Netherlands; 4 Department of Internal Medicine, Division of Endocrinology, Erasmus University Medical Center, 3015 GD Rotterdam, The Netherlands; e.vandervalk@erasmusmc.nl (E.S.v.d.V.); e.vanrossum@erasmusmc.nl (E.F.C.v.R.); 5 Department of Pathology & Medical Biology, University Medical Center Groningen, University of Groningen, 9700 RB Groningen, The Netherlands; b.van.der.vegt@umcg.nl

**Keywords:** hair analysis, hydrocortisone, cortisone, physiological stress, neoplasm, metabolic syndrome

## Abstract

**Simple Summary:**

Although animal models have suggested that chronic stress can induce cancer, epidemiological findings have been inconsistent. One of the possible reasons for this inconsistency is the challenge to measure chronic stress. Recently, hair cortisol and its inactive form cortisone have shown to be a potential biomarker for chronic stress. Our study aims to investigate the relation between chronic biological stress, as measured by hair cortisol and hair cortisone, and cancer incidence, and adjust for other factors that can impact this relation such as metabolic syndrome components in a population-based cohort. While hair cortisone was related to cancer incidence when accounting for age as a confounder and gender as a moderator, we did not observe the association with hair cortisol. The involvement of metabolic syndrome components in the relation between chronic stress and cancer initiation was not found.

**Abstract:**

Epidemiological results on the link between chronic stress and cancer initiation have been inconsistent. This study examined the relation between chronic biological stress, indicated as hair cortisol (HairF) and hair cortisone (HairE), and cancer incidence, adjusting for metabolic syndrome (MetS) components. We analyzed HairF and HairE samples from 6341 participants from the population-based cohort Lifelines in 2014. A linkage with the Dutch Nationwide Pathology Databank (Palga) provided the cancer incidence from 2015 to 2021. The association between dichotomized HairF and log-transformed HairE (LogHairE) and cancer incidence was estimated using Cox regression. MetS components were evaluated as confounders or moderators. Of the 2776 participants with known HairF levels and no cancer history, 238 developed cancer. The HairF level did not predict cancer incidence (HR: 0.993, 95%CI: 0.740–1.333). No confounders or moderators were identified. Among the 4699 participants with known HairE levels and no cancer history, 408 developed cancer. There was no association between LogHairE and cancer incidence (HR: 1.113, 95%CI: 0.738–1.678). When including age as a confounder and gender as a moderator, LogHairE was statistically significantly associated with cancer incidence (HR: 6.403, 95%CI: 1.110–36.92). In a population-based cohort, chronic biological stress, measured by HairE, was associated with cancer incidence, after controlling for age and gender.

## 1. Introduction

Cancer is regarded as one of the leading causes of death around the world [1]. A growing body of animal and clinical research supports that chronic stress is involved in the initiation, progression, and metastasizing of cancer [2,3,4]. Molecular and cellular studies reported that the alteration of glucocorticoid production by chronic stress might induce cancer development through p53 deficiency, a tumor suppressor gene [5], or immunosenescence, which can result in impaired immune surveillance against cancer [6]. In addition, there is evidence for tumor-type specific glucocorticoid production-induced pathway alterations in breast cancer [7] and colitis-associated cancer [8].

However, the findings of epidemiological studies about the association between chronic stress and cancer initiation have not been consistent [9]. Several previous studies have reported that self-reported chronic stress was linked to an increased cancer risk [10,11,12]. Contrary, other studies failed to find this association [13,14]. Meanwhile, a study in Denmark emphasized that higher everyday stress is associated with a lower risk of breast cancer [15]. The explanation for the inconsistency in former epidemiological studies might be that measuring chronic stress has been challenging due to the lack of robust measuring methods or that other characteristics, such as metabolic syndrome components, can obscure the impact of chronic stress on cancer incidence.

Chronic stress is mostly measured by different self-reported questionnaires. Such measures can be influenced by subjective aspects; thus, an objective biomarker to quantify the degree of chronic stress would be helpful. In the stress response, glucocorticoids such as cortisol and cortisone are key hormones produced by the hypothalamic pituitary adrenal axis (HPA axis) [16]. Glucocorticoids have been measured in scalp hair which potentially captures hormones secretion over periods of several months or even years [17,18], minimizing the impact of the circadian rhythm or temporary increases due to short-term stress [19]. Hair cortisol is now widely accepted as a useful biomarker of chronic stress [17,20]. The assessment of hair cortisone in parallel to hair cortisol may give even more insight into the systemic cortisol in the body [21]. Hair cortisone (HairE) might be more readily measurable than hair cortisol since hair cortisone levels are approximately 3–4-fold higher than hair cortisol (HairF), and may be less prone to disturbances from the environment such as exogenous corticosteroid use [22]. In addition, cortisone concentrations showed less individual variation than cortisol concentrations [23]. Vanaelst et al. found that HairE was associated with stressful events over the past six months among young children [24]. Therefore, HairE may also hold merit as a better biomarker for chronic stress compared to HairF.

Another possible reason for why the relation between chronic stress and cancer incidence remains unclear in epidemiological research is that other factors such as metabolic syndrome components are not taken into consideration. Previous studies revealed a positive association between metabolic syndrome components, such as abdominal obesity [25], insulin resistance [26], dyslipidemia [27], and elevated blood pressure [28,29], and the risk of cancer. In addition, the literature has illustrated that the chronic activation of the HPA axis during prolonged stress, together with behavioral changes due to stress such as physical inactivity and an unhealthy diet, can contribute to visceral fat accumulation, impaired glucose tolerance, and abnormalities of the lipid metabolism [30].

The present study aims to investigate the relation between chronic stress, as measured in HairF and HairE levels, and cancer incidence, and adjusting for demographic parameters as well as for metabolic syndrome (MetS) components, in a consecutive series of participants included in a population-based cohort.

## 2. Materials and Methods

### 2.1. Study Design

A consecutive case series from Lifelines, a multi-disciplinary prospective population-based cohort study examining the health and health-related behaviors of over 167,000 residents from the three Northern provinces in The Netherlands (Drenthe, Groningen, and Friesland), was included. Starting in 2006, participants were invited to visit a Lifelines research site once every five years to collect biological samples such as blood and scalp hair and measure BMI and blood pressure. Moreover, participants were asked to complete a structured questionnaire covering general health and lifestyle every 1.5 years [31].

For the present study, the incidence of cancer (either solid, including skin cancers, or hematological) was derived from a linkage between Lifelines cohort study and Dutch Nationwide Pathology Databank (Palga), comprising most of the pathology reports [32,33]. Palga diagnoses are in line with the International Classification of Diseases 10th Revision (ICD-10) [32,33,34]. Palga provides overviews of all new histologically or cytologically confirmed malignant tumors. In the Palga database, an individual can have several pathology reports pertaining to the diagnosis of cancer(s) [35].

### 2.2. Study Population

**Figure 1:**
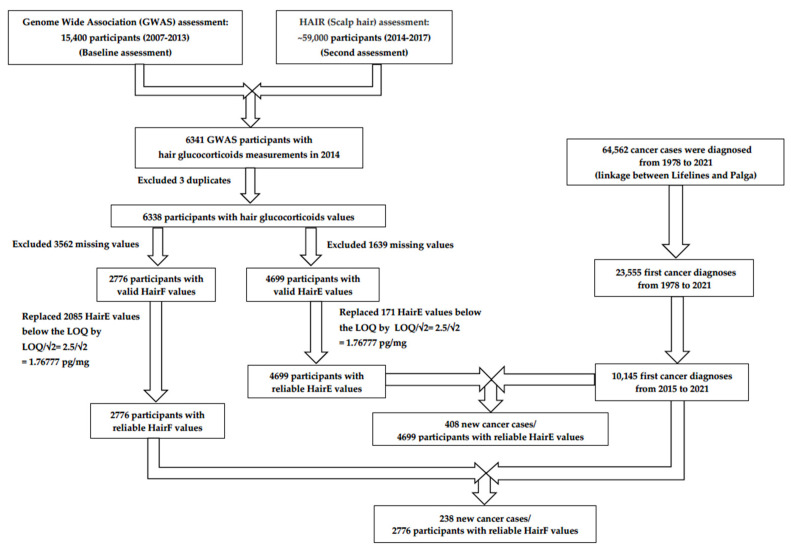
Flow chart of hair glucocorticoids and cancer incidence analyses.

Hair samples were collected in the second assessment of Lifelines. From participants with hair samples (N = 59,000), the current cohort included those who visited in 2014 and was included in the Genome-Wide Association Assessment (GWAS) to be able to relate phenotype to genotype, leaving 6341 participants with cortisol and cortisone measurements in scalp hair [36]. Although there were additional hair samples from participants, conducting hair sample analysis for 50,000 individuals was a laborious task. Therefore, we prioritized individuals who had their hair samples collected in 2014 and for whom GWAS data were available. The flowchart showing the inclusion of participants is presented in Figure 1.

### 2.3. Variables

To determine hair glucocorticoids, hair samples were collected from all participants who gave consent and had at least 3 cm of hair following standard operating procedures. For each participant, a lock of approximately 100–150 hairs from the posterior vertex as close to the scalp as possible was collected. Hair samples were taped on paper, then placed in envelopes, clearly marked and registered, and stored in the dark at room temperature. The proximal 3 cm was used for the assessment of the cortisone (HairE) and cortisol (HairF) levels, which is assumed to reflect the three-month period before hair samples were collected given a hair growth rate of 1 cm per month. Subsequently, the hair was weighed, washed, and extracted with methanol. We measured glucocorticoids in these hair samples using liquid chromatography–tandem mass spectrometry (LC-MS/MS) as previously described [37,38]. To assure qualitative measurements, we excluded hair samples weighing less than 7.5 mg, where there was an issue in sample preparation, sample matrix and/or chromatography, or where mass-spectrometry yielded no reliable peak or an aberrant ion-ratio, or co-elution. The lower limit of quantification for both hair cortisol and cortisone in our assay is currently regarded 2.5 pg/mg, and the minimum signal-to-noise ratio was set at 10.

Hair cortisol and hair cortisone measurements were carried out on the same samples simultaneously. Starting with 6341 participants with HairF and HairE measurements, 3 duplicates were excluded (Figure 1). Regarding HairF analyses, 3562 missing values (due to the requirement of a minimum amount of hair and the implementation of a cut-off value for signal-to-noise ratio) were removed, leaving 2776 participants with valid HairF values (Figure 1). Single imputation was performed by replacing 2085 HairF values below the limit of quantitation (LOQ) by LOQ/√2 = 2.5/√2 = 1.76777 pg/mg [39] (Figure 1). HairF was used as a dichotomized variable, including the values under 2.5 pg/mg and the values from 2.5 pg/mg and above. In terms of HairE analyses, 1639 missing values were excluded, resulting in 4699 participants with valid values. For the purpose of single imputation, 171 HairE values below the LOQ were replaced with LOQ/√2 = 2.5/√2 = 1.76777 pg/mg (Figure 1). Since HairE levels were not normally distributed, these levels were logarithmically transformed (LogHairE) to achieve a normal distribution. Characteristics of included participants and excluded participants for HairF and HairE analyses are shown and compared in Supplementary Tables S1 and S2.

The main outcome was cancer incidence (either solid, including skin cancers, or hematological cancer). Participants with a history of cancer or with a cancer diagnosis within one year from hair glucocorticoids measurement since 2014 were excluded. An overview of the variables that were used as possible confounders or moderators is presented in Supplementary Table S4 [40,41,42,43,44].

### 2.4. Statistical Analysis

To compare the included and the non-included participants and to describe the relation between the characteristics of the included participants and cancer incidence, Chi-square testing and independent-samples t-testing were applied.

To compare HairE levels based on participants’ characteristics, Mann–Whitney U testing and Krusal–Wallis H testing were performed. To determine the relation between HairE levels and age of study population, Spearman’s correlation was undertaken.

To assess the association between HairF/LogHairE levels and cancer incidence, Cox hazard proportional regression analysis was employed. The definition of time to cancer is described in Supplementary (Table S4). To check the assumption of proportionality for Cox regression analyses, the interaction term of time by HairF and LogHairE levels was included in the Cox hazard proportional regression. The proportional hazard assumption was not violated for HairF or LogHairE levels. Hazard ratios (HRs) and 95% confidence intervals (95%CIs) were reported.

To investigate the role of age (per 10 years), gender, metabolic syndrome components (hypertension, diabetes, dyslipidemia, and BMI classification), and health behaviors (smoking and alcohol drinking) in the relation between HairF/LogHairE levels and time to develop cancer, confounder and moderator analyses were performed. For confounder analysis, we estimated the association between candidate confounder and HairF/LogHairE levels as well as the association between candidate confounder and the time to cancer status. Subsequently, both HairF/LogHairE levels and candidate confounder were included in the relation with the time to cancer status. If the coefficient of HairF/LogHairE levels in relation to cancer in the bivariable model altered 10% or above compared to in the univariate model, we considered the candidate variable to be a confounder. For moderator analysis, bivariable Cox regression including the interaction term between HairF/LogHairE levels and each candidate moderator was examined. If the term was statistically significant, the interaction term was included in the final model. Furthermore, a stratified analysis was performed.

Multivariable Cox regression was performed including HairF/LogHairE levels and identified confounders/moderators. A *p*-value less than 0.05 was considered statistically significant. 

The data were analyzed using SPSS (Statistical Package for the Social Sciences) version 28.0 (Armonk, NY, USA) [45].

## 3. Results

A total of 2776 participants with reliable hair cortisol (HairF) levels were included in the analysis (Table 1). Their mean age was 52.9 years (SD: 10.1 years) and the majority (76.5%) were female. Of the participants, 75.1% had HairF levels below the lower limit of quantification (2.5 pg/mg). Hypertension was the most frequently observed MetS component (39.0%), followed by dyslipidemia (35.2%), abdominal obesity (16.6%), and impaired glucose tolerance (5.1%). Never-smokers accounted for 42.8% of the study population, while 37.6% and 18.1% were ex-smokers and current smokers, respectively. More than half of the participants (52.6%) were alcohol drinkers. In total, 8.6% of the study population developed cancer in the follow-up period.

A total of 4699 participants with reliable hair cortisone (HairE) levels were included in the analysis (Table 2). Similar to the study population of HairF, the mean age of the participants was 53.1 years (SD: 10.1 years), and 77.1% of them were female. Hypertension was the most frequently observed MetS component (38.5%), followed by dyslipidemia (35.5%), obesity (17.3%), and diabetes (5.2%). Never-smokers accounted for 42.0% of the study population, while 38.1% and 18.7% were ex-smokers and current smokers, respectively. More than half of the participants (52.2%) were alcohol drinkers. The median of HairE levels was 5.52 pg/mg (Q1–Q3: 4.19–7.62 pg/mg). Male participants showed significantly higher HairE levels than female participants (*p* < 0.001). Old age was associated with higher HairE levels (r_s_ = 0.09, *p* < 0.001). Elevated HairE levels were seen in individuals with hypertension, diabetes, and dyslipidemia in comparison to those without these conditions (*p* < 0.001). Current smokers demonstrated greater levels of HairE than never-smokers and ex-smokers (*p* < 0.001). Drinkers had higher levels of HairE compared to non-drinkers (*p* < 0.001).

Around 8.7% of the study population with reliable HairE levels developed cancer in the follow-up period (Table S3). Participants with cancer were, on average, four years older than those who did not develop cancer (*p* < 0.001). The proportion of cancer diagnoses was different between never-smokers and ex-smokers (*p* = 0.01). The remaining characteristics of the study population were not statistically significantly associated with cancer incidence.

The univariate Cox hazard proportional regression analysis revealed no association between the hair cortisol (HairF) level and cancer incidence (HR: 0.993; 95%CI: 0.740–1.333) (Table 3). Additionally, MetS components did not play a role as confounders or moderators in the relation between the HairF level and cancer incidence.

In the univariate Cox hazard proportional regression analysis, the log-transformed hair cortisone levels (LogHairE) were not associated with cancer incidence (HR: 1.113; 95%CI: 0.738–1.678) (Table 4). We found that age was a confounder (HR: 1.464; 95%CI: 1.334–1.607), whereas gender was a moderator in the relation between LogHairE levels and cancer incidence (HR: 2.610; 95%CI: 1.095–6.221). After taking into account age and gender, there was a statistically significant association between LogHairE levels and cancer incidence (HR: 6.403; 95%CI: 1.110–36.924) (Table 4). In the model controlling for age and stratifying by gender, LogHairE levels were not statistically significantly associated with cancer incidence among either males or females (Table 5). In addition, the impact of MetS components as confounders or moderators on the association between LogHairE levels and cancer incidence was not seen.

## 4. Discussion

To our knowledge, this is the first large-population-based cohort, utilizing high-quality data, which has investigated the relation between chronic biological stress, as measured by hair glucocorticoids, and cancer incidence. The current study did not observe a relation between hair cortisol and cancer incidence, while the association between hair cortisone and cancer incidence was found when age and gender were taken into account. Metabolic syndrome components did not influence the relation between chronic biological stress and cancer incidence in this cohort.

We found that 8.7% of our study population developed cancer from 2015 to 2021. According to Klijs et al., the prevalence of cancer in the Lifelines cohort study from 2006 to 2013 was 6.9% [46], so cancer incidence in our study was somewhat higher. The possible explanation for that may be due to the fact that the participants were slightly older in the current study compared to Klijs et al.’s study.

The relationship between hair cortisone and cancer incidence in our findings is consistent with previous studies which reported the association between chronic psychological stress and cancer initiation. A prospective cohort study in Japan found that persistently high perceived stress was significantly linked to an elevated overall cancer incidence [11]. In a Finnish cohort study, the accumulation of life events was related to an increased risk of breast cancer [12]. On the other hand, a study in Denmark emphasized that high levels of everyday stress was associated with a lower incidence of breast cancer among middle-aged women [15]. However, our results contrast with other studies that found no association between work stress [13] or chronic stressors [14] and cancer incidence. The discrepancy between the findings from our study and these studies may be explained by the fact that chronic psychological stress in these studies was measured by self-report questionnaires [11,12,15], where our study assessed chronic biological stress by hair glucocorticoids levels. Such questionnaires may not always be related to hair glucorticoids [47,48,49].

In addition, as cancer is not a single disease [50], the mechanism of how chronic biological stress contributes to the development of cancer may vary per cancer type. Basic research has shown the mechanism of how chronic biological stress induces cancer initiation for specific cancers such as breast cancer [7] and colitis-associated cancer [8]. Former studies examined the incidence of overall types of cancer [11] or of a specific type of cancer, such as breast cancer [12,15], whereas the current study included the overall cancer incidence of both solid, including skin cancers, and hematological cancers. Due to the small number of cancer cases, no analysis for particular tumor types was possible in this study.

In our study, age acted as a confounding variable since its impact on both HairE levels and cancer development might alter the actual relationship between HairE levels and cancer incidence. Age has been a strong predictor of HairF and HairE levels among participants, as evidenced by Stalder et al. in which HairF and HairE levels both increased with age [22] and Davison et al. who documented the association between older age and cortisone concentrations [51]. Increased glucocorticoids during the aging process are related to elevated levels of psychosocial stress, impaired cognitive performance, and the atrophy of memory-related regions in the brain such as the hippocampus [52]. Age is also one of the most well-known risk factors of cancer [53]. Therefore, the presence of age may influence an individual’s susceptibility to both long-term biological stress and the initiation of cancer. Hence, controlling for age is essential to accurately determine the impact of long-term biological stress on cancer occurrence.

The association between HairE levels and cancer incidence in males was approximately twice as high as in females in the present study. However, this difference was not statistically significant, possibly due to the low number of males. There is a distinction between men and women in terms of their hypothalamic–pituitary–adrenal axis reactivity to stress, with men showing a greater stress hormone response compared to women [54,55]. Also, previous investigations have indicated that men had larger quantities of hair glucorticoids compared to women [21,22,47,56,57,58]. Nevertheless, several studies reported no significant differences in hair glucocorticoids between men and women [18,59,60]. Therefore, further research will be required to determine whether a gender differences in hair glucocorticoids exists and what mechanisms may be at work.

Cortisol and cortisone are commonly known as stress response hormones, produced in response to various forms of physical or psychological stress [16]. In addition to stress, other factors that can influence systemic cortisol levels include the glucocorticoid gene [61], inflammation [62], medications use (corticosteroids), food with a high glycemic index [63], chronic pain [64], sleep deprivation [65], alcohol [66], and night-eating syndrome [67]. Cortisol is converted into the inactive form as cortisone by the enzyme11-beta-hydroxysteroid dehydrogenase type 2 (11β-HSD2), and cortisone is converted back to cortisol by the enzyme 11-beta-hydroxysteroid dehydrogenase type 1 (11β-HSD1) to ensure the differential, tissue-specific effect of glucocorticoid signaling [51]. Hair cortisone analyses alongside hair cortisol analyses may provide more information on the cumulative amount of active and inactive forms of cortisol [21]. Recent data from salivary research suggested that salivary cortisone may provide a closer and more robust reflection of systemic cortisol levels than salivary cortisol [68]. Therefore, HairE may also be a better biomarker for chronic stress compared to HairF, with the potential utility to assess long-term systemic cortisol exposure [21,22]. Moreover, several studies have explored determinants of HairE in adults and found that they are largely comparable to those of HairF [21,22,69]. A systematic review and meta-analysis of 120 manuscripts revealed a consistent positive association between hair glucocorticoids and anthropometric measurements. This link was particularly strong and clinically significant for HairE [70].

Our study has several important strengths. Firstly, the current study was embedded in Lifelines, which is a prospective population-based cohort study with a large sample size with many standardized measurements, including hair glucocorticoids for a subgroup. Secondly, the linkage between Lifelines and Palga provides the appropriate identification of the incidence of solid and hematological cancers through histological or cytological confirmed malignant tumors; thus, cancer incidence could be determined. Thirdly, indicators for MetS components were derived from high-quality data of blood samples such as fasting glucose and triglycerides, and physical measurements such as blood pressure and BMI, thereby minimizing the risk of measurement bias in these indicators.

However, there are some limitations in the present study. Initially, the current data have approximately 56.2% of missing values of HairF and 25.9% of missing values of HairE. Data on HairF and HairE were missing due to technical issues and an insufficient amount of hair for the analyses. Also, like other hair glucocorticoid studies, we have an over-representation of females in our study, as males may more often be bald or have little hair. As males are more susceptible to develop cancer at most sites, such as the bladder, kidney, colorectum, liver, esophagus, head and neck, brain, skin, and blood, compared to females [69,71], a low proportion of male participants with reliable hair glucocorticoids can weaken the relation between chronic biological stress and cancer incidence. Additionally, people with valid hair glucocorticoids were younger than those with invalid values. Participants who were current smokers had less frequently valid hair glucocorticoids compared to never-smokers. Also, individuals with hypertension and diabetes had less-often measured HairE values. Meanwhile, age, smoking, and alcohol drinking are well-known risk factors for cancer [1], and MetS components can increase cancer risk [25,26,27,28]. This may have weakened the association that we found between chronic biological stress and cancer incidence.

Another limitation is that most indicators for MetS components were (partly) collected at an earlier time point (the first assessment) than the time of measuring hair glucocorticoids (the second assessment) due to their data availability. The earlier information about MetS components may have changed between the moment of measurement and the time when hair glucocorticoids were assessed (approximately four years later); thus, it could affect the estimation of the proportion of MetS components. However, the proportion of missing information from the physical examination in the second assessment was low (2.6% at the highest) and was reduced to 0% after additionally using the information in the first assessment, while 97.4% of cases in the first assessment and the second assessment would have led to the same MetS components classification. Therefore, we do not expect that our findings would have been very different if we would have been able to use medication use information from the second assessment instead of the first assessment.

Furthermore, participants were followed for only six years in the present study, while in most previous studies, the follow-up period was over 10 years [11,12,13,15]. Considering that most cancers have a long latency period, a longer follow-up time may be needed to detect the association between chronic biological stress and cancer incidence in this cohort. While the median age of a cancer diagnosis is 66 years [72], the mean age of participants in our study was 53 years old, so a longer follow-up may also lead to more robust results.

Our first recommendation for future research is extending the follow-up period to at least 10 years which enables the clarification of the relation between long-term biological stress and cancer incidence. Secondly, as different types of cancer have distinct etiologies, future studies should explore the association between long-term biological stress and the incidence of specific types of cancer. For example, breast cancer and colitis-associated cancer should be taken into consideration because the specific mechanisms of the relation between glucocorticoids and these types of cancer were described in former studies [7,8,73]. Third, the difference between our study and other studies using self-reported measures of stress indicate that perhaps multiple dimensions of stress measurements should be taken into account to generate a complete picture. Finally, although we did not observe the role of MetS components in the relation between chronic biological stress and cancer incidence, previous studies have indicated effects of MetS components in both chronic biological stress and cancer incidence. Therefore, future research may delve deeper into the effects of MetS on this association.

The present study points towards a relation between long-term biological stress and cancer incidence, which could encourage further research to explore the causal relationship between stress and cancer, taking age and gender into consideration. Additionally, our findings could offer insights into stress reduction and management strategies that can be incorporated into cancer prevention and treatment. More precisely, it would allow for the identification of individuals with a heightened susceptibility to cancer by assessing their levels of hair glucocorticoids. These findings can be applied to implement personal screening and preventative interventions aimed at mitigating chronic biological stress levels in high-risk populations. Hence, healthcare providers may develop stress assessments as part of regular health evaluations.

## 5. Conclusions

In summary, our study revealed, in a population-based cohort, that there is an association between chronic biological stress, as indicated by hair cortisone levels, and cancer incidence. This association was observed when controlling for age as a confounder and gender as a moderator. We did not observe a role of metabolic syndrome components in this association.

## Figures and Tables

**Table 1:** Baseline characteristics of the participants in the study, overall and by hair cortisol (HairF) level (N = 2776).

Characteristics		Total		<2.5 pg/mg		≥2.5 pg/mg		*p*-Value
		N	%	N	%	N	%	
Total		2776	100	2085	75.1	691	24.9	
Gender	Male	653	23.5	462	70.8	191	29.2	**0.003 ^a^**
	Female	2123	76.5	1623	76.4	500	23.6	
Age (Mean ± SD) (years)		52.88 ± 10.08		53.00 ± 10.02		52.53 ± 10.27		0.284 ^b^
Hypertension	No	1693	61.0	1293	76.4	400	23.6	0.054 ^a^
	Yes	1083	39.0	792	73.1	291	26.9	
Diabetes	No	2634	94.9	1989	75.5	645	24.5	**0.034 ^a^**
	Yes	142	5.1	96	67.6	46	32.4	
Dyslipidemia	No	1799	64.8	1365	75.9	434	24.1	0.204 ^a^
	Yes	977	35.2	720	73.7	257	26.3	
Body mass index	Normal	1158	41.7	857	74.0	301	26.0	0.209 ^a^
	Overweight	1152	41.5	885	76.8	267	23.2	
	Obesity	462	16.6	340	73.6	122	26.4	
Smoking	Never-smokers	1189	42.8	901	75.8	288	24.2	0.610 ^a^
	Ex-smokers	1043	37.6	781	74.9	262	25.1	
	Current smokers	502	18.1	369	73.5	133	26.5	
Alcohol drinking	Non-drinkers	1315	47.4	1009	76.7	306	23.3	0.061 ^a^
	Drinkers	1461	52.6	1076	73.6	385	26.4	
Cancer	No	2538	91.4	1906	75.1	632	24.9	0.970
	Yes	238	8.6	179	75.2	59	24.8	

Body mass index was missing for 4 (0.1%) participants, while smoking status was missing for 42 (1.5%) participants. ^a^: Chi-squared test. ^b^: Independent samples *t*-test. Numbers in bold represent statistical significance.

**Table 2:** Hair cortisone (HairE) levels’ distribution according to participants’ characteristics (N = 4699).

Characteristics		Total		HairE Levels (pg/mg)					*p*-Value
		N	%	P05	P25	Median	P75	P95	
Total		4699	100	2.69	4.19	5.52	7.62	15.00	
Gender	Male	1077	22.9	3.45	5.14	6.72	9.59	19.60	**<0.001 ^a^**
	Female	3622	77.1	2.61	4.02	5.20	7.10	12.95	
Age (Mean ± SD) (years)		53.09 ± 10.07		r_s_ = 0.09					**<0.001 ^c^**
Hypertension	No	2889	61.5	2.65	4.07	5.34	7.30	13.43	**<0.001 ^a^**
	Yes	1810	38.5	2.83	4.44	5.79	8.17	17.23	
Diabetes	No	4455	94.8	2.68	4.17	5.48	7.55	14.72	**<0.001 ^a^**
	Yes	244	5.2	3.46	4.77	6.25	9.59	23.42	
Dyslipidemia	No	3029	64.5	2.68	4.14	5.37	7.37	13.70	**<0.001 ^a^**
	Yes	1670	35.5	2.73	4.77	5.77	8.18	17.23	
Body mass index	Normal	1997	42.5	2.66	4.14	5.49	7.45	13.43	0.073 ^b^
	Overweight	1883	40.1	2.77	4.27	5.60	7.68	14.48	
	Obesity	814	17.3	2.67	4.18	5.47	8.11	20.33	
Smoking	Never-smokers	1973	42.0	2.75	4.17	5.45	7.43	13.90	<0.001 ^b,^*
	Ex-smokers	1788	38.1	2.67	4.18	5.44	7.54	14.23	
	Current smokers	877	18.7	2.60	4.36	5.92	8.28	19.00	
Alcohol drinking	Non-drinkers	2246	47.8	2.73	4.11	5.30	7.29	13.55	**<0.001 ^a^**
	Drinkers	2453	52.2	2.67	4.30	5.75	7.96	16.03	

BMI was missing for 5 (0.1%) participants, while smoking status was missing for 61 (1.3%) participants. P05: 5th Percentile; P25: 25th Percentile; P75: 75th Percentile; P95: 95th Percentile. ^a^: Man-Whitney U test. ^b^: Krusal Wallis H test. ^c^: Spearman’s correlation. *: Never-smoker–Current smokers: <0.01; Ex-smokers–Current smokers: <0.01. Numbers in bold represent statistical significance.

**Table 3:** The association between hair cortisol level and cancer incidence in univariate Cox regression model.

Variables	Univariate Model	
	HR	95%CI
HairF level	0.993	0.740–1.333

**Table 4:** The association between logarithm of hair cortisone levels and cancer incidence in univariate and multivariable Cox regression models.

Variables	Univariate Model		Multivariable Model *	
	HR	95%CI	HR	95%CI
LogHairE	1.113	0.738–1.678	6.403	**1.110–36.92**
Age by 10 years			1.468	**1.337–1.611**
Gender			2.748	**1.144–6.604**
LogHairE*Gender			0.343	**0.130–0.908**

* After the assessment of age, gender, hypertension, diabetes, dyslipidemia, body mass index, smoking, and alcohol drinking. Numbers in bold represent statistical significance.

**Table 5:** The association between logarithm of hair cortisone levels and cancer incidence among male and female participants.

Variables	Males		Females	
	HR	95%CI	HR	95%CI
LogHairE	2.051	0.874–4.813	0.791	0.484–1.292
Age by 10 years	1.962	**1.623–2.373**	1.339	**1.202–1.491**

* After the assessment of age, gender, hypertension, diabetes, dyslipidemia, body mass index, smoking, and alcohol drinking. Numbers in bold represent statistical significance.

## Data Availability

The data generated in this study are available from the Lifelines cohort study upon reasonable request. For access to the data used in this study, the Lifelines research office can be contacted via www.lifelines.nl/researcher (accessed on 28 September 2023).

## Supplementary Materials

The following supporting information can be downloaded at: https://www.mdpi.com/article/10.3390/cancers16112044/s1. Table S1. The relation between valid and missing hair cortisol (HairF) values; Table S2. The relation between valid and missing hair cortisone (HairE) values; Table S3. Baseline characteristics of the participants with hair cortisone measurements, overall and stratified by cancer; Table S4. Description of variables.
