# Supplementary material for: Chronic Stress Related to Cancer Incidence, including the Role of Metabolic Syndrome Components
Source: Cancers (Basel). 2024 May 28;16(11):2044. doi: 10.3390/cancers16112044 (PMC11171137; doi:10.3390/cancers16112044)
Supplement: Supplementary file 1 [file cancers-16-02044-s001.zip › cancers-2993794-supplementary.pdf]

SUPPLEMENT

Table S1. The relation between valid and missing hair cortisol (HairF) values

Characteristics		Valid HairF values		Missing HairF values		P-value
		N	%	N	%	
**Total**		**2,776**	**43.8**	**3,562**	**56.2**	
Gender	Male	653	41.5	922	58.5	**0.031**[a]
	Female	2,123	44.6	2,640	55.4	
Age (Mean±SD) (years)		52.88±10.08		54.11± 10.40		**<0.001**[b]
Hypertension	No	1,693	44.0	2,152	56.0	0.644[a]
	Yes	1,083	43.4	1,410	56.6	
Diabetes	No	2,634	44.0	3,346	56.0	0.105[a]
	Yes	142	39.7	216	60.3	
Dyslipidemia	No	1,799	43.9	2,295	56.1	0.757[a]
	Yes	977	43.5	1,267	56.5	
Body weight	Normal	1,158	43.3	1,517	56.7	0.186[a]
	Overweight	1,152	45.1	1,405	54.9	
	Obesity	462	42.0	638	58.0	
Smoking	Never smokers	1,189	45.8	1,409	54.2	**0.018**[a*]
	Ex-smokers	1,043	42.7	1,398	57.3	
	Current smokers	502	41.4	711	58.6	
Alcohol drinking	Non-drinkers	1,315	44.8	1,620	55.2	0.134[a]
	Drinkers	1,461	42.9	1,942	57.1	
Cancer	No	2,538	44.1	3,221	55.9	0.170[a]
	Yes	238	41.1	341	58.9	

[a]: Chi-square test

[b]: Independent samples t-test

*: Never smokers - Current smokers: 0.034

Table S2. The relation between valid and missing hair cortisone (HairE) values

Characteristics		Valid HairE values		Missing HairE values		P-value
		N	%	N	%	
**Total**		4,699	74.1	1,639	25.9	
Gender	Male	1,077	68.4	498	31.6	**<0.001[a]**
	Female	3,622	76.0	1141	24.0	
Age (Mean±SD) (years)		53.09±10.07		54.95±10.76		**<0.001**
Hypertension	No	2,889	75.1	956	24.9	**0.024[a]**
	Yes	1,810	72.6	683	27.4	
Diabetes	No	4,455	74.5	1,525	25.5	**0.008[a]**
	Yes	244	68.2	114	31.8	
Dyslipidemia	No	3,029	74.0	1,065	26.0	0.706[a]
	Yes	1,670	74.4	574	25.6	
Body weight	Normal	1,997	74.7	678	25.3	0.701[a]
	Overweight	1,883	73.6	674	26.4	
	Obesity	814	74.0	286	26.0	
Smoking	Never smokers	1,973	75.9	625	24.1	**0.023[a*]**
	Ex-smokers	1,788	73.2	653	26.8	
	Current smokers	877	73.2	336	27.7	
Alcohol drinking	Non-drinkers	2,246	76.5	689	23.5	**<0.001[a]**
	Drinkers	2,453	72.1	950	27.9	
Cancer	No	4,291	74.5	1,468	25.5	**0.034[a]**
	Yes	408	70.5	171	29.5	

[a]: Chi-square test

[b]: Independent samples t-test

*: Never smokers - Current smokers: 0.048

Table S3. Baseline characteristics of the participants with hair cortisone meassurements, overall and stratified by cancer (N=4,699)

Characteristics		Total		No cancer		Cancer		P-value
		N	%	N	%	N	%	
**Total**		**4,699**	**100**	**4,291**	**91.3**	**408**	**8.7**	
Gender	Male	1,077	22.9	989	91.8	88	8.2	0.497[a]
	Female	3,622	77.1	3,302	91.2	320	8.8	
Age (Mean±SD) (years)		53.09±10.07		52.73±9.98		56.89±10.20		**<0.001[b]**
Hypertension	No	2,889	61.5	2,656	91.9	233	8.1	0.057[a]
	Yes	1,810	38.5	1,635	90.3	175	9.7	
Diabetes	No	4,455	94.8	4,071	91.4	384	8.6	0.511[a]
	Yes	244	5.2	220	90.2	24	9.8	
Dyslipidemia	No	3,029	64.5	2,763	91.2	266	8.8	0.745[a]
	Yes	1,670	35.5	1,528	91.5	142	8.5	
Body mass index	Normal	1,997	42.5	1,802	90.2	195	9.8	0.064[a]
	Overweight	1,883	40.1	1,737	92.2	146	7.8	
	Obesity	814	17.3	749	92.0	65	8.0	
Smoking	Never smokers	1,973	42.0	1,827	92.6	146	7.4	**0.035[a*]**
	Ex-smokers	1,788	38.1	1,614	90.3	174	9.7	
	Current smokers	877	18.7	798	91.0	79	9.0	
Alcohol drinking	Non-drinkers	2,246	47.8	2,042	90.9	204	9.1	0.351[a]
	Drinkers	2,453	52.2	2,249	91.7	204	8.3	

BMI was missing for 5 (0.1%) participants, while smoking status was missing for 61 (1.3%) participants.

[a]: Chi-squared test

[b]: Independent samples t-test

*: Never smokers - Ex-smokers: 0.031

Table S4. Description of variables

Variables		Definition	Time points of data collection
Age per 10 years		Age of each participant was divided by 10 (years)	Age: the second assessment
Gender		Male or Female	Gender: the second assessment
Metabolic syndrome components	Hypertension	Yes: if Systolic Blood Pressure (SBP) ≥140 mm Hg and/or Diastolic Blood Pressure (DBP) ≥90 mm Hg [1] and/or use of antihypertensive medication based on Anatomical Therapeutic Chemical (ACT) codes: C02 (antihypertensives), C03 (diuretics), C07 (b-blocking agents), C08 (calcium channel blockers) and C09 (agents acting on the renin-angiotensin system) [2].<br>No: if not the above cases.	Systolic Blood Pressure (SBP) and Diastolic Blood Pressure (DBP): the first assessment and the second assessment<br>Medication: the first assessment
	Diabetes	Yes: if a participant who had self-reported diabetes or fasting blood glucose ≥ 7.0 mmol/L or glycated hemoglobin (HbA1c) ≥ 6.5% [3] or use of medication for diabetes treatment regarding ACT codes: A10A (Insulin and analogues), and A10B (Blood glucose lowering drugs, excl Insulin) and A10X (Other drugs used in diabetes) [2].<br>No: if not the above cases.	Fasting blood glucose and glycated hemoglobin (HbA1c): the first assessment and the second assessment<br>Medication: the first assessment
	Dyslipidemia	Yes: if triglycerides >1.7 mmol/L (150 mg/dL) or high-density lipoprotein (HDL) cholesterol < 1.0 mmol/L (40 mg/dL) in male, or < 1.3 mmol/L (50 mg/Dl) in female [4]; or use of medication for dyslipidemia treatment, including C10AA (Statin), C10AB (Fibrates), C10AC (Bile acid sequestrants), C10AD (Nicotinic acid), C10AX06 (n-3 fatty acids), C10AX09 (Ezetimibe), C10AX13 (PCSK9 inhibitor: Evolucumab), C10AX14(Alirocumab), C10AX17 (Evinacumab) [2].<br>No: if not the above cases.	Triglycerides and high-density lipoprotein (HDL) cholesterol: the first assessment and the second assessment<br>Medication: the first assessment
	Body weight	Normal weight if body mass index (BMI) <25 kg/m², overweight if BMI ≥ 25–30 kg/m², and obesity if BMI ≥ 30 kg/m² [4].	Body mass index (BMI): the second assessment

Table S4. Description of variables (continue)

Variables		Definition	Time point of data collection
Health behaviors	Smoking	Smoking was categorized into never smokers (those who had never smoked and had also never smoked for longer than a year), ex-smokers (those who had smoked for more than a year but had stopped smoking for at least one month at the time of data collection) and current smokers (those who reported that they were current smokers or had stopped smoking less than a month) [5].	Smoking: the first assessment.
	Alcohol drinking	Alcohol intake was evaluated by the Lifelines 110-item food-frequency questionnaire (FFQ), which reported the total amount of alcohol in grams per day that a participant consumed in the previous month. Participants were defined as non-drinkers when they reported drinking < 1 g/day in the previous month or drinkers when they reported ≥ 1 g/day in the previous month.	Alcohol intake: the first assessment
Cancer incidence		Cancer: if participants without of history of cancer have a new cancer diagnosis (either solid or hematological cancer).<br>No cancer: if participants did not have a new cancer diagnosis.	From 2015 to 2021
Time (years) to cancer status		The time from the begining of the study to the first cancer diagnosis.	Since the exact date of hair collection was not available, we counted time from Jan 1st, 2015, because all participants visited in 2014. Time (years) to cancer diagnosis was computed by subtracting this date from the date of diagnosis, while we subtracted this date from Jan 26, 2021, for participants without cancer since this was the date of PALGA record retrieval.

REFERENCES

1. Williams B, Mancia G, Spiering W, Agabiti Rosei E, Azizi M, Burnier M, Clement DL, Coca A, de Simone G, Dominiczak A, et al. 2018 ESC/ESH Guidelines for the management of arterial hypertension. *Eur Heart J* **2018**, *39*(33):3021-104.

2. ATC/DDD Index. Available online: https://www.whocc.no/atc_ddd_index/ (access on 21 February 2022).

3. Cosentino F, Grant PJ, Aboyans V, Bailey CJ, Ceriello A, Delgado V, Federici M, Filippatos G, Grobbee DE, Hansen TB, et al. 2019 ESC Guidelines on diabetes, pre-diabetes, and cardiovascular diseases developed in collaboration with the EASD. *Eur Heart J* **2020**, *41*(2):255-323.

4. Catapano AL, Graham I, De Backer G, Wiklund O, Chapman MJ, Drexel H, Hoes AW, Jennings CS, Landmesser U, Pedersen TR, et al. 2016 ESC/EAS Guidelines for the Management of Dyslipidaemias. *Eur Heart J* **2016**, *37*(39):2999-3058.

5. Faruque MO, Vonk JM, Bültmann U, Boezen HM. Airborne occupational exposures and inflammatory biomarkers in the Lifelines cohort study. *Occupational and Environmental Medicine* **2021**, *78*(2):82.
